# Beyond phase shifting: targeting circadian amplitude for light interventions in humans

**DOI:** 10.1093/sleep/zsae247

**Published:** 2024-10-21

**Authors:** Oliva Walch, Franco Tavella, Jamie M Zeitzer, Renske Lok

**Affiliations:** Department of Neurology, University of Michigan, Ann Arbor, MI, USA; Arcascope Inc, Arlington, VA, USA; Department of Biophysics, University of Michigan, Ann Arbor, MI, USA; Department of Computational Medicine and Bioinformatics, University of Michigan, Ann Arbor, MI, USA; Department of Psychiatry and Behavioral Sciences, Stanford University, Stanford, CA, USA; Mental Illness Research Education and Clinical Center, VA Palo Alto Health Care System, Palo Alto, CA, USA; Department of Psychiatry and Behavioral Sciences, Stanford University, Stanford, CA, USA

**Keywords:** amplitude, lighting, interventions, mathematical modeling, translational

Circadian rhythms, intrinsic biological oscillations synchronized to the 24-hour day–night cycle, govern a myriad of physiological processes in living organisms [1–3]. These rhythms are essential for maintaining human health and well-being by regulating sleep–wake cycles, hormone secretion, metabolic activity, and cognitive function. Among the concepts relevant to rhythms are **phase** (the timing of a specific point in the cycle), **period** (the duration of one complete cycle), and **amplitude** (the extent of variation within the cycle or the “strength” of the rhythm). Numerous human experiments have examined how circadian phase and period interact with environmental cues [4–7]. Among these environmental cues, light holds unparalleled significance as the primary synchronizer of circadian rhythms [8], with photoreceptive cells in the retina relaying information from the external light–dark cycle to the brain’s suprachiasmatic nucleus (SCN), the central pacemaker that coordinates the timing of various physiological processes.

Disruptions to circadian rhythms have been implicated in various human disorders, including sleep disturbances [9], mood disorders [10], metabolic dysfunction [11], and impaired immune responses [12]. Consequently, interventions targeting circadian rhythms have gained attention as potential therapeutic strategies. However, the landscape of circadian interventions is complex, encompassing a wide array of approaches that can be interpreted differently among researchers and practitioners. Traditional circadian interventions often involve manipulating the timing of light exposure, focusing on classic phase advancing and phase-delaying mechanisms [13–15], such that early morning light exposure is used to advance circadian rhythms, while evening light exposure is used to delay them [4–7]. Yet there are a curious and growing number of associative studies in which individuals are exposed to lighting at *other* times of day, during which there is little impact of light on the circadian phase (the so-called circadian “dead zone” or “dead point” in humans), have shown improvements in outcome measures, suggesting alternative circadian mechanisms may be at play [16, 17].

Such results prompt attention back to seminal experiments of the 1990s that established both phase and amplitude as crucial components of the circadian framework [18]. Light pulses were shown, in a phase- and amplitude-dependent manner, to either enhance or suppress circadian amplitude as represented by the relative amplitude of the first harmonic of a three-harmonic fit of core body temperature (CBT) data obtained during constant routines [18]. Data from these experiments played a crucial role in refining mathematical models of circadian rhythms, but despite their significance, the amplitude effects observed in these early studies have been largely overlooked in real-world circadian health interventions.

This research letter explores the theoretical implications of timed light exposure on circadian amplitude through the lens of mathematical models. We propose that the benefits of timed light exposure may arise not only from shifts in circadian timing but also from enhancements in the circadian amplitude of the central pacemaker. We furthermore echo the calls of others to standardize circadian amplitude measurements and emphasize the need for more research focused on circadian amplitude [19].

## Theoretical Implications for Amplitude as an Intervention Target

Mathematical models can provide valuable insights into hypotheses about light-induced enhancements in circadian amplitude in humans. Various limit cycle oscillator models of the circadian clock [18, 20, 21] incorporate concepts of both phase and amplitude, which are crucial for explaining specific empirical data, including Type 0 resetting [4]. These concepts enable the creation of phase response curves, amplitude response curves, and phase–amplitude resetting maps. These can provide a visual means to convey theoretical predictions derived from circadian resetting models, even in the absence of experimental data to validate these predictions. While some mathematical models [18, 20–22] use phenomenological notions of amplitude (e.g. the distance of coordinates in a modified van der Pol oscillator from the singularity), more recent models [21] explicitly define amplitude, often denoted as *R*, as the coherence of firing in the SCN. In other words, highly synchronized firing represents higher amplitude values, whereas poor synchronization (indicating a “low confidence” in the clock’s phase estimate) corresponds to lower amplitude [23–26].

**Figure 1.**
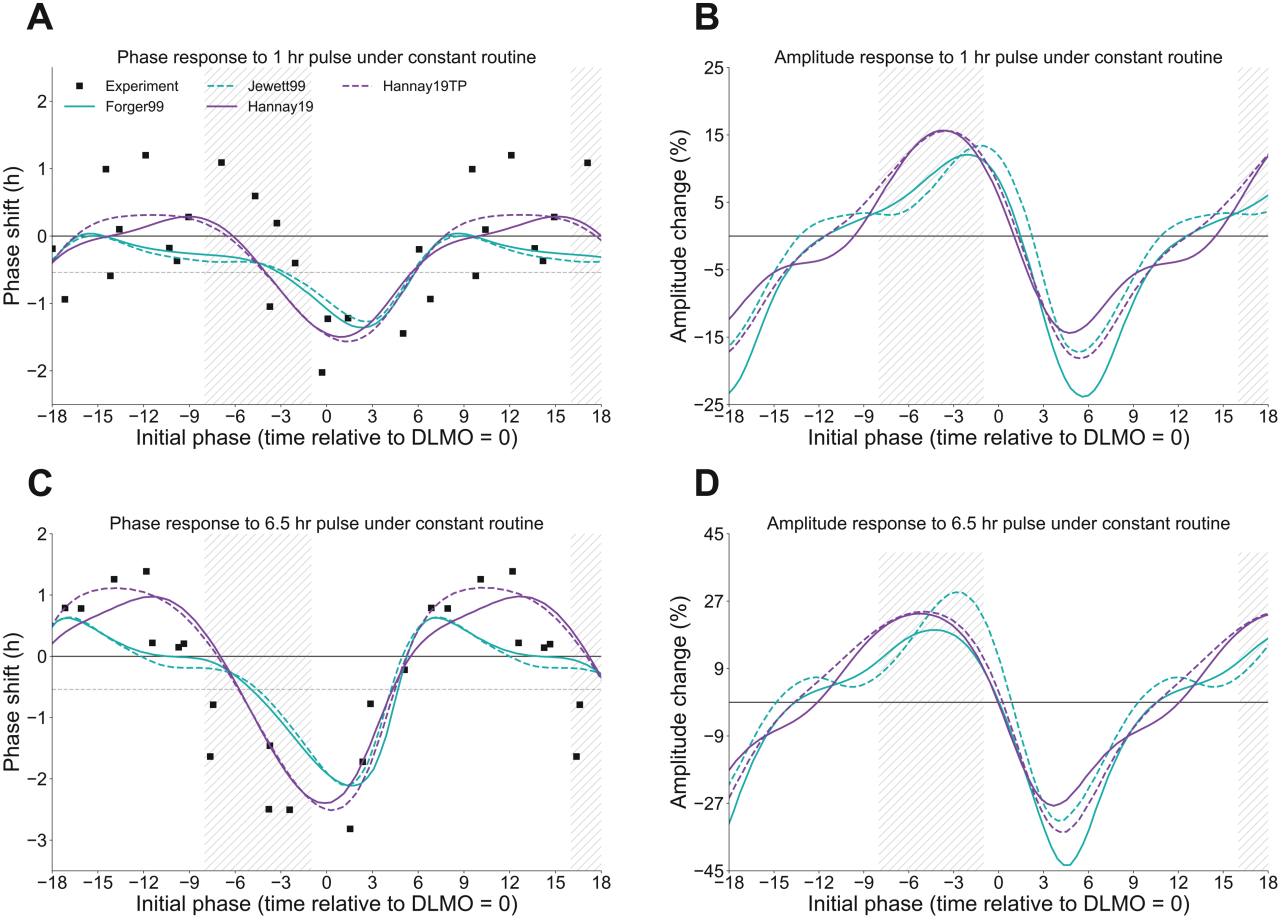
Comparing phase and amplitude response curves for four different models of the human circadian clock. (A) Phase response curve data (black squares) to a 1-h pulse of bright light (8000 lux, estimated ~7232 melanopic lux) [27] under constant routine conditions [6] and model fits from [20–22]. Phase shift curves are adjusted for the natural drift of the clock (–0.54 h, gray horizontal dashed line). (B) The predicted percent amplitude change for the same models and experiment as in A. (C) Phase response curve data (black squares) to a 6.5-h pulse of bright light under constant routine conditions [28] and model fits from [20–22]. (D) Predicted percentage amplitude change for the same models and experiment as in C. DLMO-8 to DLMO-1 is highlighted in all plots as a reasonable range for afternoon/evening (extending from 1:00 pm to 8:00 pm for a person whose habitual DLMO is at 9:00 pm). While differences exist in the peaks of amplitude response in B and D, with the more classical van der Pol oscillator models [20, 22] peaking later than alternate models [21], a general trend of amplitude boosting in the hours before DLMO and amplitude suppression at DLMO + 6 (approximately CBTmin) can be readily observed. Phase shifts and amplitude percent changes are calculated immediately at the end of the light pulse by comparing simulations with and without stimuli. For Forger99 [20] and Jewett99 [22] models, the amplitude is calculated as the two-norm of state variables x and x_c_, while for Hannay19 models [21], the amplitude is taken directly from the *R* variable that explicitly models this quantity.

When using these models to calculate how the human central circadian system responds to light, there are minor variations in the exact timing of peaks and troughs in terms of phase shifting and amplitude-changing properties (Figure 1). However, a consistent trend emerges: (1) light in the early morning or evening can induce changes in the timing of the circadian system without altering circadian amplitude and act as an anchor point, (2) exposure to light during the day’s “dead zone” tends to boost amplitude, (3) and light close to the unmasked core body temperature minimum (CBTmin) tends to suppress amplitude. Interventions aimed at enhancing amplitude may thus prioritize seeking midday light while actively avoiding light during the late night and early morning hours (Figure 1).

Circadian responses to light appear to become rapidly saturated, an important consideration when evaluating daytime lighting interventions. Given continuous light of the same brightness, the drive to the circadian clock initially peaks at a higher value before dropping to a lower steady-state value and remaining there [29]. As such, daytime light interventions may be more blunted in their overall effect than morning light interventions, in which the system is maximally able to respond to light after a prolonged period of darkness (i.e. sleep) [30, 31]. This implies that circadian amplitude effects could be more pronounced in individuals who generally live under more dimly lit circumstances, in alignment with studies linking increased daytime light exposure to better sleep in older individuals [32–34].

Yet emerging experimental evidence suggests that amplitude effects may persist even in the presence of such photic saturation. Model-derived definitions of low amplitude have been associated with diabetes risk [35] and weight gain [36]. In both cases, the studied populations (the UK Biobank subject pool and a cohort of school-age children, respectively) would not be expected to live in habitually dim environments. Moreover, brighter daytime light has been found to correlate with higher daytime firing rates in the SCN in a diurnal mammal [37]. This SCN-level definition of amplitude, similar to how it is defined in physiological mathematical models [21], likely represents the “truest” sense of amplitude in the central pacemaker. However, while such a definition is unequivocally rigorous, it is challenging to reproduce in humans. We revisit the concept of practical definitions for amplitude in section “Practical Limitations of Amplitude as an Intervention Target.”

## Evidence for Maximizing Circadian Amplitude Through Improved Sleep–Wake Stability

**Figure 2.**
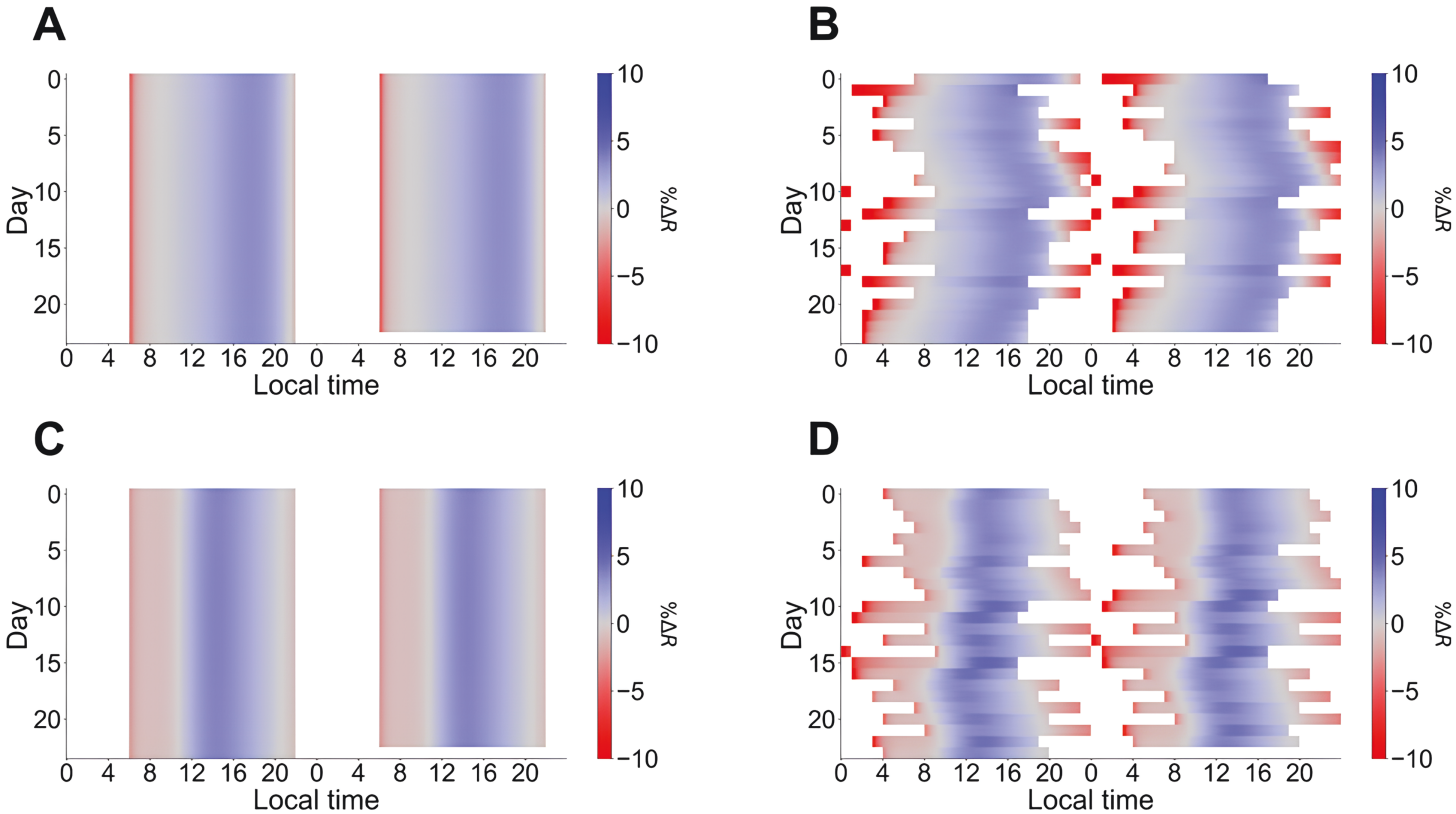
High sleep irregularity can lead to more stimulus during the amplitude suppression region of the amplitude response curve across models. Simulated actograms showing regular (Sleep Regularity Index [38] = 1.0; A and C) and irregular (Sleep Regularity Index = 0.75; B, D) sleep schedules. Color corresponds to the difference between expected light exposure on that schedule (assumed 500 lux during wake) and darkness, as captured by the percent difference in instantaneous amplitude response to light (%Δ*R*). Red periods, mainly at the beginning of the day and in the middle of the night, correspond to amplitude suppression, and blue periods, mainly during the day, correspond to amplitude boosting, with amplitude responses near zero encoded in gray. White regions correspond to expected sleep periods and darkness, during which the amplitude response on the schedule is equal to the amplitude response to darkness. A and B show responses from [20]; C and D show responses from the single population model in [21]. As sleep irregularity increases, the amount of wakefulness during the amplitude suppression part of the amplitude response curve tends to increase (more dark red exposure). While there appears to be less amplitude suppression in D (as indicated by lighter red), this is in part due to the rapid saturation of the photic response in that model.

Similar to how mathematical models can illuminate how light induces enhancements in central circadian amplitude, modeling can provide a straightforward explanation for how consistent sleep–wake routines and sleep stabilization could lead to circadian amplitude improvements (Figure 2). In individuals with stable sleep–wake timing, light exposure overlaps primarily with amplitude-boosting regions of the amplitude response curve (blue area; Figure 2, A and C). Conversely, those with irregular sleep–wake rhythms experience light exposure across more parts of the phase advance and delay zones, with light more likely to bleed into the amplitude suppression zone surrounding CBTmin (red areas; Figure 2, B and D). This suggests that irregular sleep–wake schedules could contribute to reduced circadian amplitudes.

Enhanced circadian amplitude may explain connections between sleep regularity and improved health outcomes, such as the recent report that sleep regularity predicts mortality better than sleep duration in the UK Biobank database cohort [39]. Maintaining a regular sleep schedule is associated with consistent patterns of light exposure [40], whereas irregularities in sleep timing are linked to lower daytime light levels and increased light exposure during the biological night [38]—both of which, according to literature [41, 42] and theoretical models (Figure 2, B and D), can lead to diminished circadian amplitude. Following the recent consensus statement from the National Sleep Foundation highlighting the significance of sleep regularity for health and performance [43], research into sleep regularity is likely to increase, and we would encourage theoretical circadian amplitude effects to be considered as part of that work.

Several studies hint at a potential relationship between circadian amplitude and sleep. Sleep stabilization, which minimizes light exposure that could reduce circadian amplitude (Figure 2, B and D), is associated with improved mood and sleep [44]. Increased afternoon light, which would typically increase circadian amplitude according to the phase–amplitude resetting maps [18], is associated with reduced sleep-wake fragmentation in older adults [16]. While it is intriguing to hypothesize that these effects are secondary to increased circadian amplitude, the exact causal mechanism remains uncertain. To the best of our knowledge, there are currently no published intervention studies specifically designed to boost central circadian amplitude; this represents a significant gap in the literature that future research should aim to address.

## Practical Limitations of Amplitude as an Intervention Target

Neglecting circadian amplitude as an intervention target is not merely an oversight. A “pure” definition of circadian amplitude—as it pertains to the coherence of firing in the SCN—is nearly impossible to measure experimentally in humans, necessitating the use of downstream measures. Various proxies have been used, such as concentrations of cortisol and melatonin, but these are not necessarily reflective of the central pacemaker and can readily be influenced by non-circadian factors [45]. Similarly, amplitude measures like activity can be masked in numerous ways, such as a person having an unusually active day. While considering proxies, it is critical to emphasize that such measures may not represent anything circadian at the individual level. A robust amplitude measure is likely to necessitate long data collection periods, akin to those employed in foundational phase-amplitude resetting studies, to account for the time-varying nature of the signal [46]. However, this complexity, compounded by the intricate process of measuring the circadian clock amidst masking effects, renders the assessment of circadian amplitude outside the lab challenging.

It is natural to seek an absolute definition of amplitude, similar to a 3 pg/mL threshold for determining the circadian phase through melatonin onset. Yet there is high variation in absolute levels of melatonin production among individuals, with some individuals naturally being high producers [47]. Does a high producer of melatonin have a more robust circadian clock? Possibly, but it seems equally plausible that their actual circadian amplitude is masked by some genetic trait predisposing them to make more melatonin relative to the rest of the population. Without SCN-level insights, it is impossible to know which is the case. For example, peak melatonin values are significantly higher in women than men, but women’s unmasked CBT amplitude is lower [48]. Thus, women’s “circadian amplitude” is higher by some measures, while by others it is lower. Similarly, there are differences in CBT setpoint, which is, on average, 0.3–0.7°C higher in the post-ovulatory luteal phase compared with the preovulatory follicular phase in naturally cycling women [49]. However, this does not necessarily mean that women in these phases have a higher circadian amplitude.

Beyond interindividual differences, absolute amplitude measures can also be vulnerable to questions of cause and effect. Social factors such as employment have been linked to differences in the amount of melatonin, with unemployed individuals producing more melatonin than full-time workers [50]. Women using hormonal birth control have melatonin peak values closer to men’s [50]. In addition, some medical conditions, such as Huntington’s disease [51], posttraumatic stress disorder [52], coronary artery disease [53], and Parkinson’s disease [54], are correlated with lower melatonin production. In the face of many masking influences, it is hard to imagine a single measurement that can robustly tell us if a person has high or low circadian amplitude.

**Figure 3.**
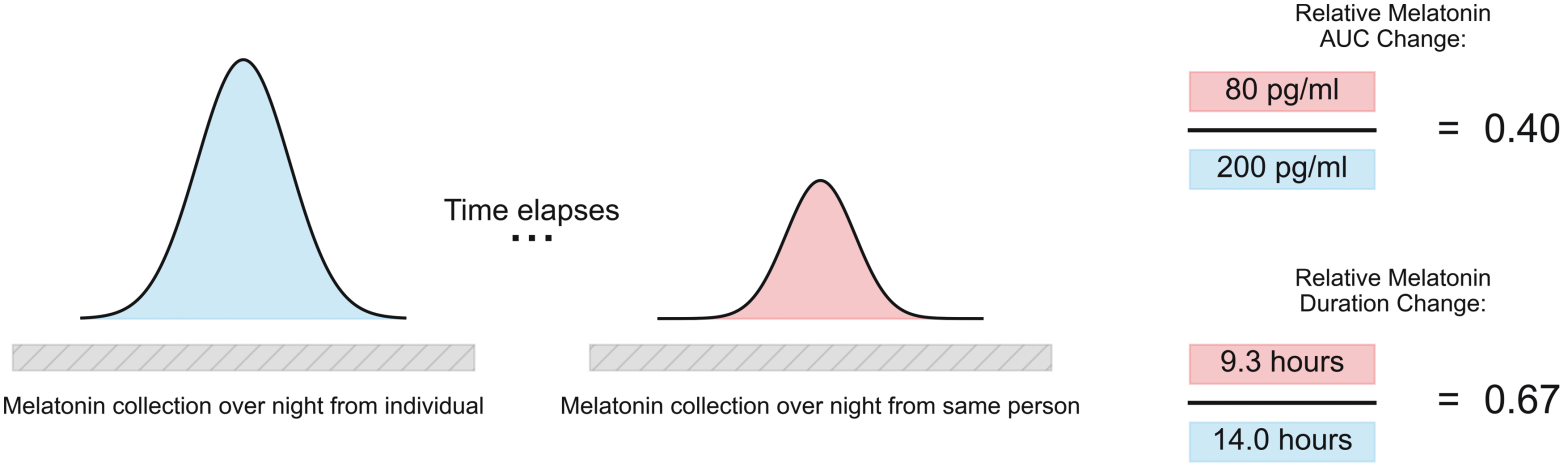
One method for quantifying relative amplitude changes. On the left is a hypothetical melatonin profile from a continuous sampling of an individual throughout the night (blue). On the right is a second hypothetical melatonin profile (red), taken at least 1 day later, i.e. after an intervention. The success or failure of an amplitude intervention could be captured by the ratio of two metrics derived from the curve, e.g. if the second area is 40% of the first area, or the width of the second is two-thirds that of the first, as in the example, amplitude suppression could be said to have occurred. The same approach could be applied to other definitions of amplitude (e.g. peak value, difference between maximum and minimum) and other unmasked, 24-hour continuous signals such as temperature or gene expression.

An alternative strategy involves assessing the efficacy of an amplitude intervention *relative* to a baseline amplitude. This approach—quantifying amplitude based on relative changes over time—could be applied to any definition of amplitude, including the peak of a signal (e.g. peak cortisol), the width of a signal (e.g. the duration of time between melatonin onset and melatonin offset), or the difference between the area under the curve (AUC) of a time-varying quantity. For example, one could conduct 24 hours of melatonin sampling at regular intervals under dim light conditions to establish the peak, the duration between onset and offset, or the AUC of melatonin for an individual at baseline. Subsequently, a second 24-hour collection post-intervention could be undertaken, with the same metrics calculated again. The relative improvement or lack thereof in amplitude could be quantified by determining the ratio between the two values (Figure 3). In this manner, addressing concerns related to an individual’s baseline melatonin production potentially masking true circadian amplitude becomes more feasible. The division process can be conceptualized as a mechanism to “cancel out” the innate variation in absolute levels of any specific marker. This approach aims to provide a more accurate evaluation of amplitude intervention effects by normalizing against individual baseline variations in melatonin production, and similar approaches could be employed in the case of temperature.

Yet numerous questions persist: Is this approach genuinely valid? Does amplitude exhibit a ceiling effect and manifest uniformly across all individuals? In instances where measurements do not cover a complete 24-hour cycle, is the appearance of low amplitude indicative of an actual decrease, or could it be attributed to sampling at inappropriate times? Additionally, how do individual differences fluctuate over time, influenced by factors such as season and the timing and content of their self-selected light diet? Are two nights of measurement adequate, or are multiple nights required to establish internal validity? Considering the broad spectrum of interindividual differences, it is crucial to carefully select control groups and utilize a within-subject design in any circadian intervention. However, this approach also impacts participants, leading us back to the fundamental question: What is your intervention trying to achieve? If the goal is to phase shift the participant, amplitude considerations may not be necessary. However, if the goal is more in line with improving the robustness of the circadian clock, questions about how best to define and quantify amplitude may be critical.

## Next Steps

To advance the conversation around circadian amplitude, it is crucial to establish a robust gold standard or consensus on valid markers for assessing relative changes in circadian amplitude in humans. Even if proposed relative approaches, like calculating the ratio between two separate 24-hour collections of melatonin duration or AUC, become accepted as definitions for amplitude change, the significant challenges and costs of conducting such measurements at scale persist. Therefore, there is an urgent need for circadian amplitude output measures that do not require prolonged laboratory stays.

Additionally, the circadian field should refocus its attention on evaluating lighting interventions not only based on their impact on phase but also considering their expected effects on central circadian amplitude. It is vital to customize interventions according to patients’ specific complaints, acknowledging the diversity of circadian challenges. For example, individuals struggling to fall asleep early might benefit from phase-shifting interventions, while those having difficulty staying asleep may find amplitude-focused interventions more appropriate. Likewise, individuals dealing with chronic daytime fatigue and insufficient nighttime tiredness could benefit from amplitude boosters rather than phase advances or delays. While enhancing circadian amplitude may hold significance for specific issues, it is unlikely to be a universal solution. For instance, in cases of jetlag, where the primary concern is the timing of the circadian clock, amplifying circadian amplitude may be counterproductive during the early parts of the phase-shifting process. Similarly, treatments for depression involving extended exposure to light throughout the night would be expected to suppress amplitude yet have proven to be effective at alleviating symptoms [55]. Moreover, we cannot ignore the positive effects of afternoon light exposure on non-circadian measures, including mood [56], when interpreting results from interventions targeting amplitude.

Emphasizing the need to move away from one-size-fits-all approaches, it is crucial to acknowledge that circadian sleep–wake disorders are complex, and patient satisfaction with treatments often varies [57]. Individualized considerations, such as delivering light at the correct circadian phase for the desired output, can significantly impact treatment outcomes. Integrating the concept of amplitude into the framework of lighting interventions may enhance precision and effectiveness in addressing circadian challenges for patients. In an era where circadian disruptions have become increasingly prevalent, understanding the delicate interplay between circadian rhythms and light is paramount. This exploration not only broadens our comprehension of circadian interventions but also underscores the importance of moving beyond a one-size-fits-all paradigm, recognizing the unique nature of individual circadian profiles in the pursuit of optimal health and well-being.

## Data Availability

The complete codebase and data are available on GitHub at: https://github.com/arcascope/amplitude-simulations
